# Biologically-Active Gold(III) Complexes

**DOI:** 10.1155/MBD.1999.271

**Published:** 1999

**Authors:** R. V. Parish

**Affiliations:** Department of Chemistry UMIST Manchester M60 1QD UK

## Abstract

A review is given of the background to and results of the succesful pharmacological testing of [AuX_2_(damp)] (X = Cl, OAc; damp = 2−Me_2_ NCH_2_C_6_ H_4_) against a range of microbes, fungi and tumouts, culminating in *in vivo* xenografts of ZR-1-75. These are the first fully evaluated gold(III) complexes. The activity and reactions of the diacetato-complex bear a resemblance to cisplatin, and some of the relevant chemistry is discussed. Preliminary screening data for C,P-chelated tertiary phosphine derivatives of gold(III) are presented.


### BIOLOGICALLY-ACTIVE GOLD(III) COMPLEXES

R. V. Parish

Department of Chemistry, UMIST, Manchester, M60 1QD, UK

### ABSTRACT

A review is given of the background to and results of the succesful pharmacological testing of $[AuX_2(damp)]$ ($X = Cl$, OAc; damp = $2\text{-}Me_2NCH_2C_6H_4$) against a range of microbes, fungi and tumours, culminating in *in vivo* xenografts of ZR-1-75. These are the first fully evaluated gold(III) complexes. The activity and reactions of the diacetato-complex bear a resemblance to cisplatin, and some of the relevant chemistry is discussed. Preliminary screening data for C,P-chelated tertiary phosphine derivatives of gold(III) are presented.

### INTRODUCTION

Given that the anti-tumor activity of platinum(II) has been investigated for over 30 years, it is surprising that gold(III) has received so little attention. The two are isoelectronic and isosteric: $d^8$ configurations and square-planar complexes; they undergo similar types of subsitution reactions.

There are two obvious problems. (*a*) The additional positive charge on the gold atom means that the ligand sets cannot be the same if the same overall charge is to be attained. Thus, for neutral complexes of the cisplatin type gold(III) would have to have three negatively charged ligands. (*b*) Compounds of gold show a distressing tendency to undergo reduction to the metal. Those who have attempted any synthetic chemistry in the field know how readily deposits of elemental gold appear.

It might be desirable to mimic some other features of active platinum compounds, *e.g.* the *cis*-disposition of two readily displaceable ligands and, perhaps, the presence of a primary or secondary amine ligand.

### PREVIOUS WORK

### N,O⁻-Systems

The above challenges are not difficult to overcome. We decided a few years ago to use chelating mono-negative ligands to force the *cis* geometry and to neutralise the third positive charge, and chose to begin with Schiff-bases and pyridine 2-carboxylates (**I**, **II**).[1] Complexes of the desired type can be obtained, albeit not without some difficulty (it is much easier to obtain the bis-chelated complexes than the mono-chelates, Equ 1). However, as soon as these materials encountered biological media, reduction to elemental gold occurred, presumably with oxidation of the cell-material and, hence, disastrous toxicity.[2]

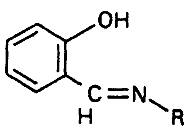

**I**

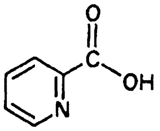

**II**

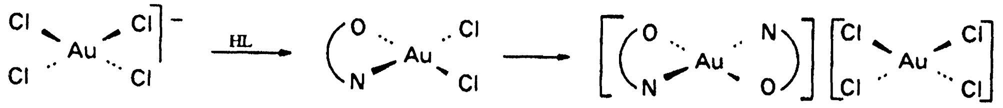

. . . (1)

### N,C⁻-Systems

It was necessary to stabilise the gold(III) centre against reduction, and we argued that this would best be done by using a softer ligand system. This would reduce the positive charge on the gold atom and make it less oxidising and less prone to reduction. Organometallics seemed to be the answer, since a _-carbon ligand is certainly soft and would serve as the anionic group, and organic derivatives of gold are fairly stable. There had been a brief report that $Ph_4As[AuCl_2Me_2]$ and $[AuMe_2(_\text{-}SCN)]_2$ inhibited P388.[3] We preferred chelate complexes, in order to be sure of the configuration, and some relevant materials had already been reported; these were derived from 2-phenylpyridine (**III**, Constable)[4] or from diemthylbenzylamine or azobenzene (**IV**, **V**, Vicente);[5,6] we decided to look at these, and their ring-substituted analogues and to prepare new complexes with related $N,C^-$ ligands (**VI-VIII**).

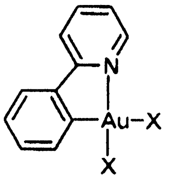

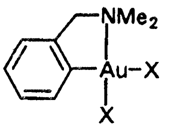

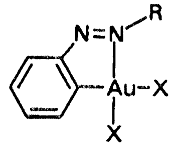

**III** **IV** **V**

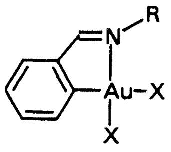

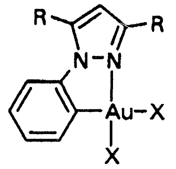

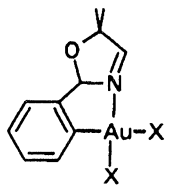

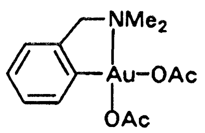

**VI** **VII** **VIII** **IX**

We used mainly the trans-metallation route *via* the mercury(II) derivative (Scheme 1).[7,8] This method works far better than the direct auration route pioneered by Constable[4] and used by others for some substituted pyridine derivatives,[9] which give rather low yields.

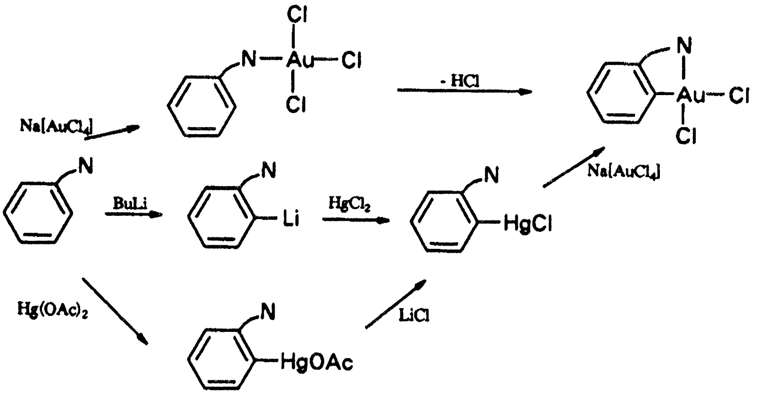

Scheme 1

TABLE I Minimum Inhibitory Concentrations (μg/mL) against Bacteria

| | Sytaphylococcus aureus | Enterococcus faecalis | Krebsiella pneumoniae | Escheria coli | Pseudomonas aeruginosa |
|---|---|---|---|---|---|
| **III** | 1.0 - 2.5 | 2.5 - 10 | 25 - 200 | 25 - 100 | 25 - 100 |
| **IV** | 1.0 - 2.5 | 1.0 - 2.5 | 10 - 25 | 10 - 25 | 10 - 25 |
| **IX** | 0.25 - 1.0 | 0.25 - 1.0 | | 2.5 - 10 | 50 - 100 |
| **X** (X = Cl) | > 100 | > 100 | > 100 | > 100 | > 100 |
| **X** (X = Br) | 10 - 25 | 25-100 | > 100 | > 100 | > 100 |
| Ciprofloxacin[a] | < 0.25 | 1.0 - 2.5 | < 0.25 | < 0.25 | < 0.25 |

[a] a quinolone antibiotic, used as standard

We now have a large number of complexes of these types, and we have done much of the conventional ligand-substitution chemistry. For instance, introduction of a tertiary phosphine may or may not lead to "unlatching" of the nitrogen donor atom (Equ. 2, 3). Adding another chelate gives more straightforward products (Equ. 4-6), except when two dithiocarbamates are introduced, when fluxional products are obtained containing one monedentate and one bidentate thiolate ligand (Equ 7).

However, the principal point of interest here is the possible biological activity of the complexes. Fortunately, Johnson Matthey had a new screening facility and were anxious to try out precious-metal complexes; we gave them complexes **III** and **IV** (X = Cl), which were put through several screens involving bacteria and fungi (Tables I and II).[11]

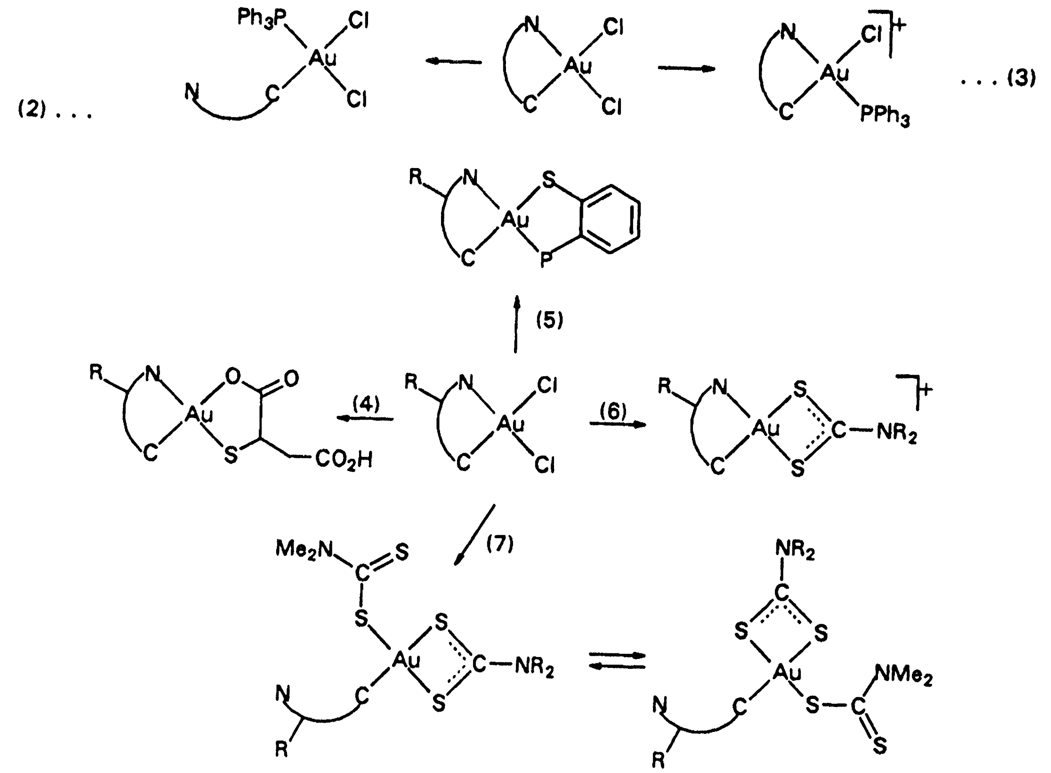

TABLE II Minimum Inhibitory Concentrations (μg/mL) against fungi

| | Candida Albicans[a] | Candida Albicans | Cryptococcus neoformans | Aspergillus niger | Aspergillus fumigatus |
|---|---|---|---|---|---|
| **III** | 25 - 100 | 25 - 100 | 1.0 - 2.5 | 25 - 100 | 25 - 100 |
| **IV** | 10 - 25 | 25 - 100 | 2.5 - 10 | 10 - 25 | 2.5 - 10 |
| **X** (X = Cl) | 25 - 100 | | 25 - 100 | 25 - 100 | 25 - 100 |
| **X** (X = Br) | 25 - 100 | | 10 -25 | 2.5 - 10 | 10 - 25 |
| Amphotericin B[a] | < 0.25 | < 0.25 | < 0.25 | 0.25 - 1.0 | 0.25 - 1.0 |

[a] a polyene antifungal, used as standard

TABLE III Comparative cytotoxicity against CHO cells (% survival)

| Conc. (μg/mL) | 100 | 10 | 1.0 | 0.1 |
|---|---|---|---|---|
| **IV** | 2.2 | 69.6 | 84.3 | 91.3 |
| **IX** | 0.4 | 96.1 | 97.9 | 99.9 |
| cisplatin | 26.2 | 89.0 | 92.9 | 96.9 |
| Ciprofloxacin | 90.0 | 101.2 | 99.9 | 96.9 |
| Amphotericin B | 2.4 | 72.7 | 88.2 | 102.3 |

TABLE IV $IC_{50}$ values (μg/mL) against human tumour lines *in vitro*

| | SW620 (colon) | SW116 (colon) | SW403 (colon) | ZR-75-1 (breast) | HT29/219 (rectum) | HT1376 (bladder) | SK-OV-3 (ovary) |
|---|---|---|---|---|---|---|---|
| **IV** | 50 | 48 | 56 | 11 | 22 | 12 | 18 |
| **IX** | 281 | 238 | | 45 | 67 | 13 | 13 |
| cisplatin | 120 | 135 | 200 | 15 | 30 | 15 | 14 |

TABLE V $IC_{50}$ values (μg/mL) for ovarian carcinomas *in vitro*

| | HX63 | CH1 | CH1-R[a] | A2780 | A2780-R[a] |
|---|---|---|---|---|---|
| **IX** | 34 | 11 | 12 | 3.5 | 35 |
| cisplatin | 18 | 0.12 | 0.56 | 1.2 | 10 |

[a] R = platinum-resistant

One is looking here for activity at reasonably low concentrations and, more particularly, for discrimination between different organisms; simple biocidal activity is not enough. At this stage, complex **III** was dropped, and the toxicity of the dimethylbenzylamine complex **IV** against Chinese Hamster Ovary cells (CHO) was compared with that of cisplatin (Table III). The gold complex was more toxic than cisplatin but, against various tumour lines *in vitro*, it showed interesting selectivity (Table IV). The highspot of the testing was the xenograft (human tumour lines grafted on to living nude mice). The profiles (Fig. 1a) were not dissimilar to those of cisplatin, although the dose levels needed were rather higher.[12]

There was a possibility that the effectiveness of **IV** was being limited by its poor aqueous solubility, and it was suggested that the diacetato analogue, **IX**, might be better. The data here were very promising indeed in anti-bacterial and CHO tests (Tables I, III) and even better against the tumour lines (Tables IV, V). Of particular interest was the toxicity against CH1, one form of which is resistant to cisplatin.[13] The xenograft tests were also superb (Fig 1b).[14]

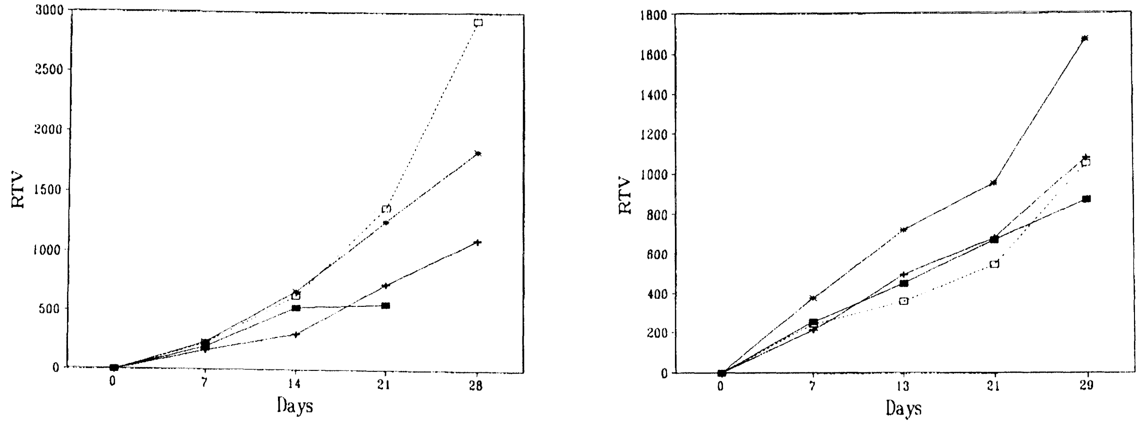

(a) (b)

Figure 1 Growth of breast tumour ZR-75-1 xenograft treated (a) with **IV** [dose levels: open square, none (control); *, 6.25 mg/kg; +, 12.5 mg/kg; ■, 25 mg/kg], and (b) with **IX** [*, 6.25 mg/kg; +, 12.5 mg/kg; ■, 25.0 mg/kg; ❐, cisplatin, 4.5 mg/kg].

Various biological mechanistic studies were carried out by Johnson Matthey[8,14] which suggested, as might be expected, some differences from the mode of action of cisplatin. Meanwhile, we at UMIST were characterising **IX** and exploring its chemistry. One oddity was that the $^{13}C$ NMR spectra showed a strange difference in intensity of the two sets of acetate peaks (Fig 2a), which we eventually attributed to a hydrolysis reaction due to traces of water in the solvents; the pure $D_2O$ solution showed a very exaggerated intentsity difference (Fig 2b). Confirmation was obtained by using bone-dry acetonitrile and deliberately adding water to it (Fig 2c,d). It was then possible to see two stages of hydrolysis, one very fast, giving rapid exchange between co-ordinated and free acetate, and one much slower. The rapid one presumably involves the acetate

*trans* to the labilising carbon ligand (Equ. 8, 9).[13] These results were of great interest, since the mechanism of action of cisplatin is thought to depend on hydrolysis.

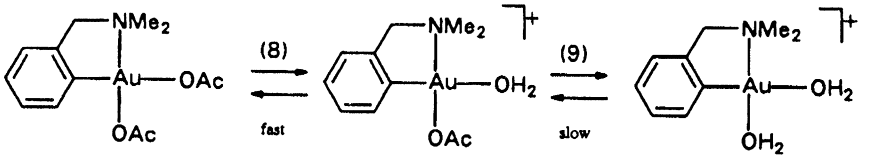

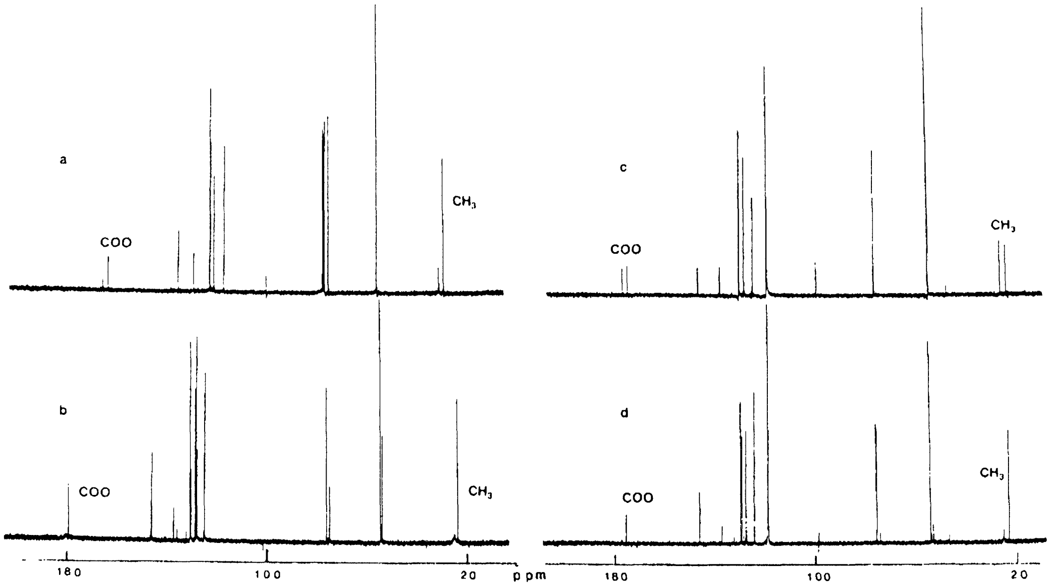

Figure 2. $^{13}$C NMR spectra of **IX**. (a) in $CDCl_3$, (b) in $D_2O$, (c) in $CD_3CN$ (dry), (d) in $CD_3CN/H_2O$ ($Au:H_2O$ = 1:20).

In aqueous or dmso solution, **IX** reacts readily with a range of biological ligands.[13] Cysteine and glutathione reacted in predictable ways, to give chelated complexes but purines and nucleosides gave various degrees of co-ordination, ranging from zero to quantitative. Co-ordination was always monodentate, and the NMR indicated various donor sites (the $NH_2$ group, or $N^1$ or $N^7$). While this is different from what is normally thought to be the case for platinum, a recent paper suggests that pH- and temperature-dependent equilibria and rearrangements may occur.[15]

Recently, workers in New Zealand have performed antimicrobial and anti-tumor screening on [Au(damp)Z] where Z is a chelating urylene or thiosalicylate ligand [$(RN)_2C$=O or 2-$SC_6H_4CO_2$; (damp = 2-$Me_2NCH_2C_6H_4$)]. The data show good toxicity and promising anti-tumour activity.[16] Clearly systems of this type bear further investigation, and we are arranging further screening tests on our gold(III) C,N-chelates. Modifications of the ligands are being made to attempt to obtain better initial solubilities.

**P,C⁻-Systems**

It would clearly be prudent to examine other ligand systems, and we picked up an old but curious report by Martin Bennett, who showed that oxidation of gold(I) complexes of diphenylstyrylphosphine gave P,C chelate systems (Scheme 2).[17] The chemistry of this system is very strange, invlvoing redistribution and redox reactions.[18] However, Johnson Matthey have performed preliminary antimicrobial screening. Activity was not very good (Tables 1,2), slightly better for the dibromo derivative, probably because it is less insoluble. Both had to be dissolved in acetone before adding water; we are currently working on synthesising more soluble derivatives, and shall try again.

### CONCLUSION

Gold(III) complexes have interesting biochemical activity provided that the ligands are chosen to stabilise the gold against reduction and to provide adequate solubility. It is certain that their mode of action will be different from that of platinum(II) systems, but this is probably an advantage in that it opens a different spectrum of activity.

**ACKNOWLEDGEMENT**

I am grateful to Dr Bill Henderson (Waikato) for supplying his data[16] in advance of publication.

**REFERENCES**

1 Dar, A., Moss, K., Cottrill, S.M., Parish, R.V., McAuliffe, C.A, Pritchard, R.G., Beagley, B., and Sandbank, J., *J. Chem. Soc., Dalton Trans*, 1992, 1907.
2 This work was done in collaboration with Professor Colin Lock.
3 Sadler, P.J., Nasr, M., Narayanan, V.L., in *Platinum Coordination Complexes in Cancer Chemotherapy* (eds. Hacker, M.P., Douple, E.B., Krakof,I.H.), Martinus Nijhoff Publishing, Boston, 1984, pp290-304.
4 Constable, E.C. and Leese, T.A., *J. Organomet. Chem.*, **363** (1989) 419.
5 Vicente, J., Chicote, M.T. and Bermúdez, M.D., *J. Organomet. Chem.*, **268** (1984) 191.
6 Vicente,J. and Chicote, M.T., *Inorg. Chim. Acta*, **54** (1981) 105.
7 Bonnardel, P.-A., Parish, R.V. and Pritchard, R.G. *J. Chem.Soc., Dalton Trans.*, 1996, 3185.
8 Parish, R.V., Howe, B.P., Wright, J.P., Pritchard, R.G., Buckley, R.G., Elsome, A.M. and Fricker, S.P., *Inorg. Chem.*, **35** (1996) 1659.
9 Cinellu, M.A., Zucca, A., Stoccoro, S., Minghetti, G., Manassero, M. and Sansoni, M., *J. Chem. Soc., Dalton Trans.*, (1995) 2865 and (1996) 4217.
10 Fuchita, Y., Ieda, H., Tsunemune, Y., Kinoshita-Nagaoka, J. and Kawano, H., *J. Chem. Soc., Dalton Trans.*, (1998) 791.
11 See also Fricker, S.P., *Metal-Based Drugs* **6** (1999), ###.
12 Parish, R.V., Howe, B.P., Wright, J.P., Pritchard, R.G., Buckley, R.G., Elsome, A.M. and Fricker, S.P., *Inorg. Chem.*, **35** (1996) 1659.
13 Parish, R.V., Mack, J., Hargreaves, L., Wright, J.P., Buckley, R.G., Elsome, A.M., Fricker, S.P., and Thobald, B.R.C., *J. Chem. Soc., Dalton Trans.*, 1996, 69.
14 Buckley, R.G., Elsome, A.M., Fricker, S.P., Henderson, G.R., Theobald, B.R.C., Parish, R.V., Howe B.P. and Kelland, L.R., *J. Med. Chem.*, **39** (1996) 5208.
15 Arpahlati, J., Klika, K.D., Sillanpää R. and Kivekas, R., *J. Chem. Soc., Dalton Trans.*, 1998, 1397.
16 Dinger,M.B. and Henderson, W., *J. Organomel. Chem.*, **557**, 231-241 (1998); **560**, 233 (1998).
17 Bennett, M.A., Hoskin, K., Kneen, W.R., Nyholm, R.S., Hitchock, P.B., Mason, R., Robertson, G. and Towl, A.C., *J. Amer. Chem. Soc.*, 1971, **93**, 4591, 4593.
18 Parish, R.V., Khan, T., Boyer, P. and Jones, A., unpublished data.

**Received: September 8, 1998 -**
**Accepted in final form: September 30, 1998**

